# Supplementary material for: ProkEvo: an automated, reproducible, and scalable framework for high-throughput bacterial population genomics analyses
Source: PeerJ. 2021 May 21;9:e11376. doi: 10.7717/peerj.11376 (PMC8142932; doi:10.7717/peerj.11376)
Supplement: Supplemental Information 1 — * SISTR is used only for Salmonella genomes. [file peerj-09-11376-s001.docx]

| **Program** | **Version** | **Description** | **Databases** | **Link** | **Reference** |
| --- | --- | --- | --- | --- | --- |
| **parallel-fastq-dump** | 0.6 | Parallel wrapper for SRA Toolkit | No | https://github.com/rvalieris/parallel-fastq-dump | [32] |
| **Trimmo-matic** | 0.38 | Trimming tool for Illumina NGS reads | No | https://github.com/timflutre/trimmomatic | [33] |
| **FastQC** | 0.11 | Tool to quality control for sequencing data | No | https://github.com/s-andrews/FastQC | [34] |
| **SPAdes** | 3.13 | Genome assembler | No | https://github.com/ablab/spades | [35] |
| **QUAST** | 5.0 | Evaluation tool for genome assembly | No | https://github.com/ablab/quast | [36] |
| **Plasmid Finder** | 2.0 | Tool for detection and characterization of plasmid sequences | Integrated curated database of plasmid replicon sequences. | https://bitbucket.org/genomicepidemiology/plasmidfinder/src/master/ | [37] |
| **SISTR*** | 1.0 | Tool for Salmonella In Silico Typing | Integrated database of cgMLST allelic profiles from Salmonella genomes. | https://github.com/phac-nml/sistr_cmd | [14] |
| **Prokka** | 1.13 | Prokaryotic genome annotation tool | Integrated set of core and HMM databases that can also be customized. | https://github.com/tseemann/prokka | [38] |
| **Roary** | 3.12 | Pan-genome and core-genome alignment tool | Can use user-specified Kraken database for quality control. | https://github.com/sanger-pathogens/Roary | [39] |
| **fastbaps** | 1.0 | Improved version of the BAPS clustering method | No | https://github.com/gtonkinhill/fastbaps | [40] |
| **MLST** | 2.16 | Tool for multilocus-sequence typing | Integrated set of PubMLST databases for multiple organisms that can also be customized. | https://github.com/tseemann/mlst | [41] |
| **ABRicate** | 1.0 | Tool for screening of contigs for AMR and virulence genes | Integrated set of DNA sequence databases such as NCBI, CARD, ARG_ANNOT, Resfinder, VFDB, that can also be specified by the user. | https://github.com/tseemann/abricate | [43] |
| **GNU Bash** | 4.1.2 | Unix shell and command tool | No | https://www.gnu.org/software/bash/ |  |
