# Supplementary material for: ProkEvo: an automated, reproducible, and scalable framework for high-throughput bacterial population genomics analyses
Source: PeerJ. 2021 May 21;9:e11376. doi: 10.7717/peerj.11376 (PMC8142932; doi:10.7717/peerj.11376)
Supplement: Supplemental Information 2 — While the raw files are available on FigShare, the intermediate files include additional filtering used to produce the corresponding Figures. [file peerj-09-11376-s002.docx]

|  | **Link** |
| --- | --- |
| **Main project** | https://figshare.com/projects/ProkEvo/78612 |
| **SRA identifications for *S.* Typhimurium genomes** | https://doi.org/10.6084/m9.figshare.13601198 |
| **SRA identifications for *S.* Infantis genomes** | <https://doi.org/10.6084/m9.figshare.13601192> |
| **SRA identifications for *S.* Newport genomes** | <https://doi.org/10.6084/m9.figshare.13601195> |
| **SRA identifications for *S*. *aureus* genomes** | <https://doi.org/10.6084/m9.figshare.13601189> |
| **SRA identifications for *C*. *jejuni* genomes** | <https://doi.org/10.6084/m9.figshare.13601186> |
| **Distribution of antibiotic resistance genes across STs of *S.* Infantis** | https://doi.org/10.6084/m9.figshare.13082906.v1 |
| **Distribution of antibiotic resistance genes across STs of *S.* Newport** | https://doi.org/10.6084/m9.figshare.13083032.v1 |
| **Distribution of antibiotic resistance genes across STs of *S.* Typhimurium** | https://doi.org/10.6084/m9.figshare.13083176.v1 |
| **Distribution of antibiotic resistance genes across 3 serovars of *S. enterica* lineage I** | https://doi.org/10.6084/m9.figshare.13082795.v1 |
| **Relative frequencies for *C. jejuni* STs** | https://doi.org/10.6084/m9.figshare.13082771.v1 |
| **Antibiotic resistance gene distribution across major STs of *C. jejuni*** | https://doi.org/10.6084/m9.figshare.13082777.v1 |
| **Relative frequencies for S*. aureus* STs** | https://doi.org/10.6084/m9.figshare.13082783.v1 |
| **Antibiotic resistance gene distribution across major STs of S*. aureus*** | https://doi.org/10.6084/m9.figshare.13082786.v1 |
| ***S*. *aureus* population structure** | https://doi.org/10.6084/m9.figshare.13601294.v1  https://doi.org/10.6084/m9.figshare.13601291.v2  https://doi.org/10.6084/m9.figshare.13601288.v1  https://doi.org/10.6084/m9.figshare.13601279.v1  https://doi.org/10.6084/m9.figshare.13601276.v1  https://doi.org/10.6084/m9.figshare.13601270.v1  https://doi.org/10.6084/m9.figshare.13601267.v2  https://doi.org/10.6084/m9.figshare.13601264.v1  https://doi.org/10.6084/m9.figshare.13601210.v1  https://doi.org/10.6084/m9.figshare.13601204.v1 |
| ***C*. *jejuni* population structure** | https://doi.org/10.6084/m9.figshare.13601255.v1  https://doi.org/10.6084/m9.figshare.13601252.v1  https://doi.org/10.6084/m9.figshare.13601246.v1  https://doi.org/10.6084/m9.figshare.13601240.v2  https://doi.org/10.6084/m9.figshare.13601231.v1  https://doi.org/10.6084/m9.figshare.13601228.v1  https://doi.org/10.6084/m9.figshare.13601225.v1  https://doi.org/10.6084/m9.figshare.13601213.v1  https://doi.org/10.6084/m9.figshare.13601207.v1  https://doi.org/10.6084/m9.figshare.13601201.v1 |
| **Statistics for runtimes of individual ProkEvo jobs for *S.* Newport and *S.* Typhimurium** | https://doi.org/10.6084/m9.figshare.13640639 |
