# Supplementary figures and images for: ProkEvo: an automated, reproducible, and scalable framework for high-throughput bacterial population genomics analyses
Source: PeerJ. 2021 May 21;9:e11376. doi: 10.7717/peerj.11376 (PMC8142932; doi:10.7717/peerj.11376)

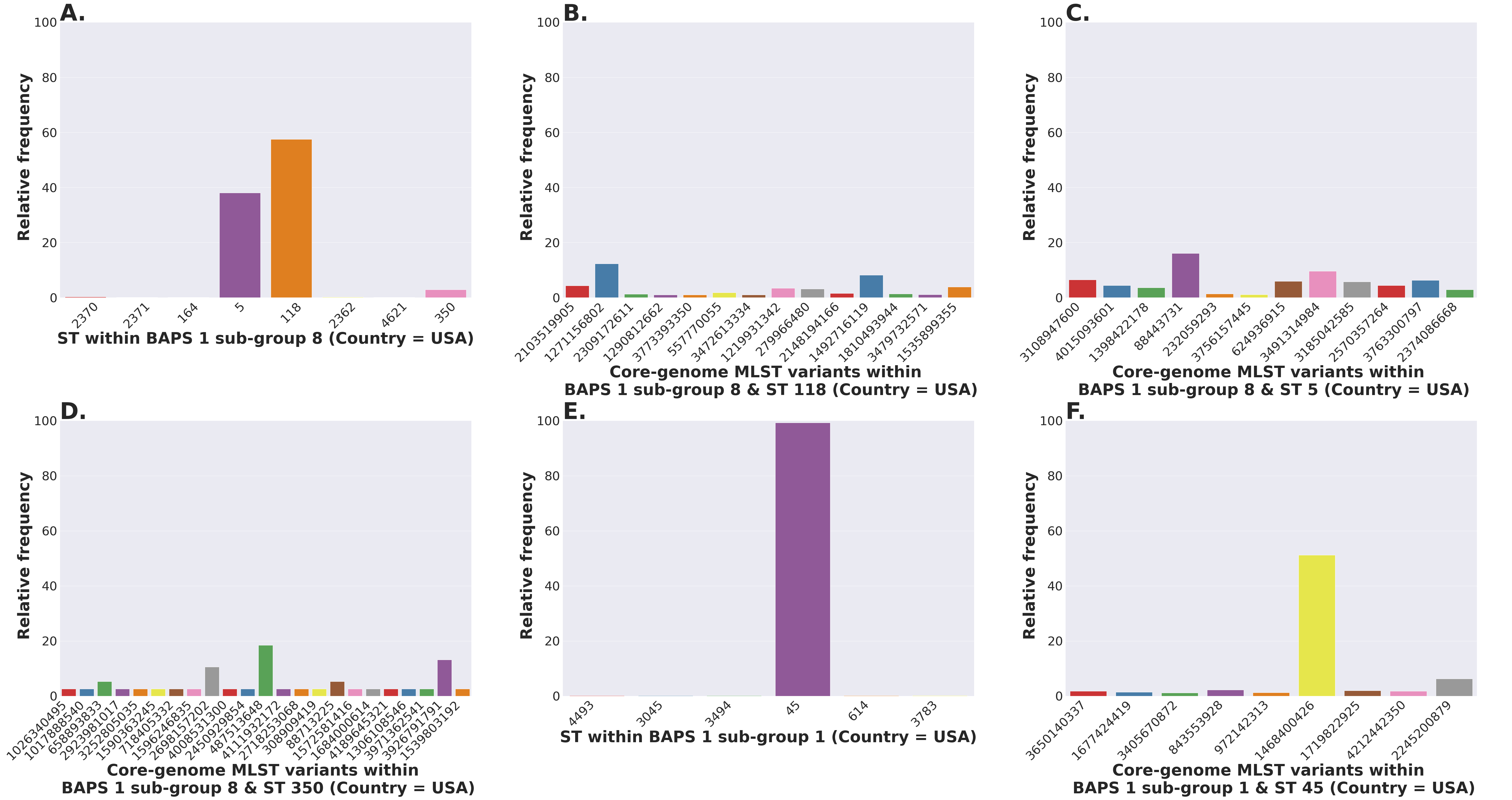

Supplement: Supplemental Information 3 — ’(A) ST distribution with BAPS1 sub-group 8 (excluding STs with relative frequency below 0.1%). (B) Core-genome MLST variant distribution within BAPS1 sub-group 8 and ST118 (excluding variants with relative frequency below 1%). (C) Core-genome MLST variant distribution within BAPS1 sub-group 8 and ST5 (excluding variants with relative frequency below 1%). (D) Core-genome MLST variant distribution within BAPS1 sub-group 8 and ST350 (excluding variants with relative frequency below 1%). (E) ST distribution with BAPS1 sub-group 1 (excluding STs with relative frequency below 0.1%). (F) Core-genome MLST variant distribution within BAPS1 sub-group 1 and ST45 (excluding lineages with relative frequency below 1%). The number of filtered genomes (i.e., genomes that passed assembly quality control metrics) used as an input in this analysis was 2,365.’ [file peerj-09-11376-s003.png]

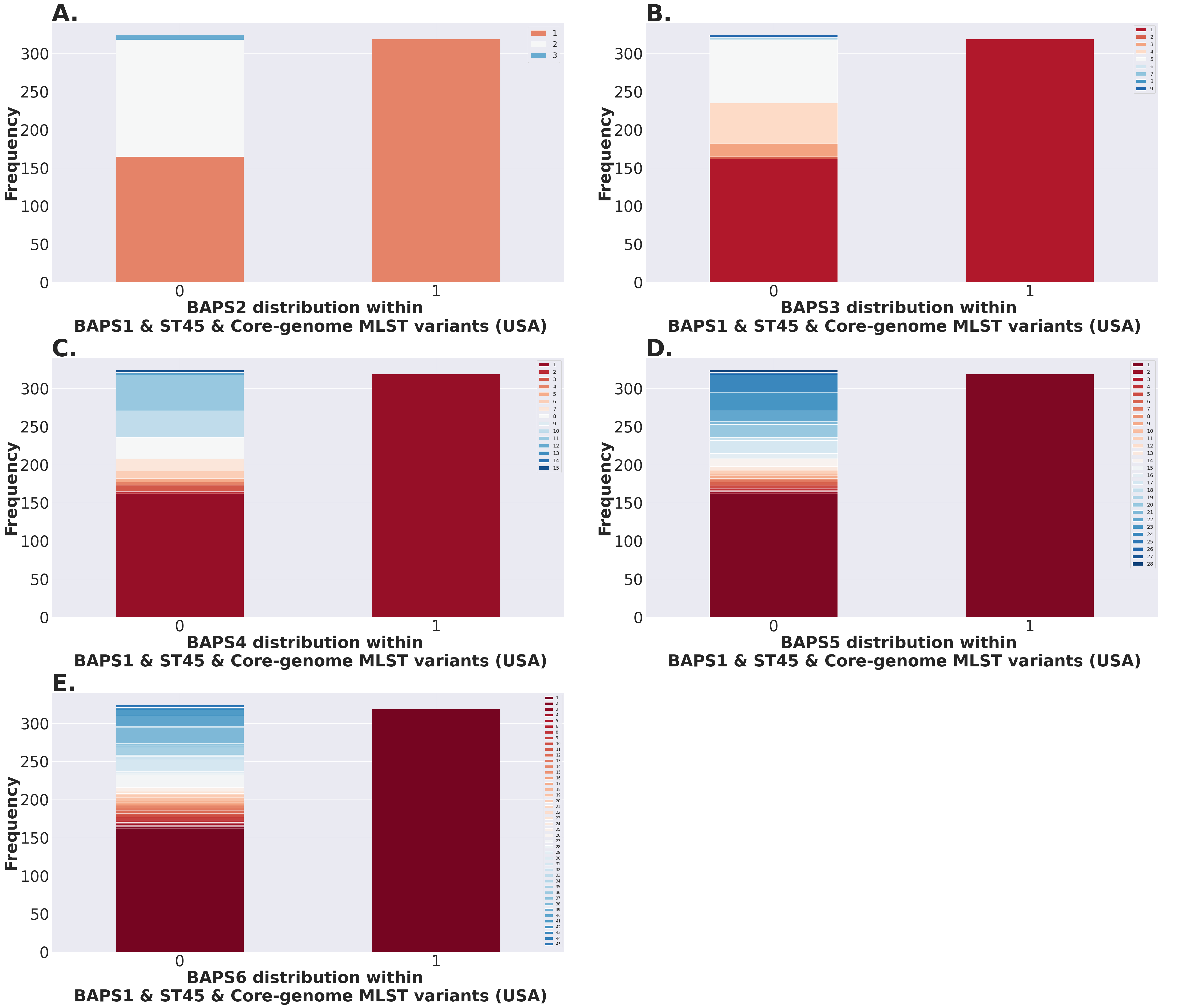

Supplement: Supplemental Information 4 — ’First, core-genome MLST (cgMLST) genotypes were classified as 1 if it were cgMLST 1468400426, and 0 otherwise. cgMLST 1468400426 can be called a major cgMLST variant (i.e., higher relative frequency variant) within BAPS1 sub-group 1 and ST45. The goal was to compare the distribution of BAPS levels 2-6 between cgMLST 1468400426 or the other cgMLST variant as a sub-population of BAPS1 sub-group 1 and ST45. (A-E) Frequency of BAPS levels 2-6, respectively, when comparing group classified as 1 (core-genome MLST variant = 1468400426) or 0 (core-genome MLST variants = others), within BAPS sub-group 1 and ST45, as part of a hierarchical approach for analysis of the S. Newport population in the United States (USA). Specifically, with this analysis one can evaluate how genotypically homogenous (i.e., clonal) the population of that variant is when compared to other cgMLSTs all combined. A highly clonal population will have few or even a single BAPS sub-group as the levels go up from BAPS2 to BAPS6. A more diverse variant will have the number of sub-groups increased, the more one stratifies the population going from BAPS2 to BAPS6. The number of quality-controlled genomes used as an input for these analyses was 2,365.’ [file peerj-09-11376-s004.png]

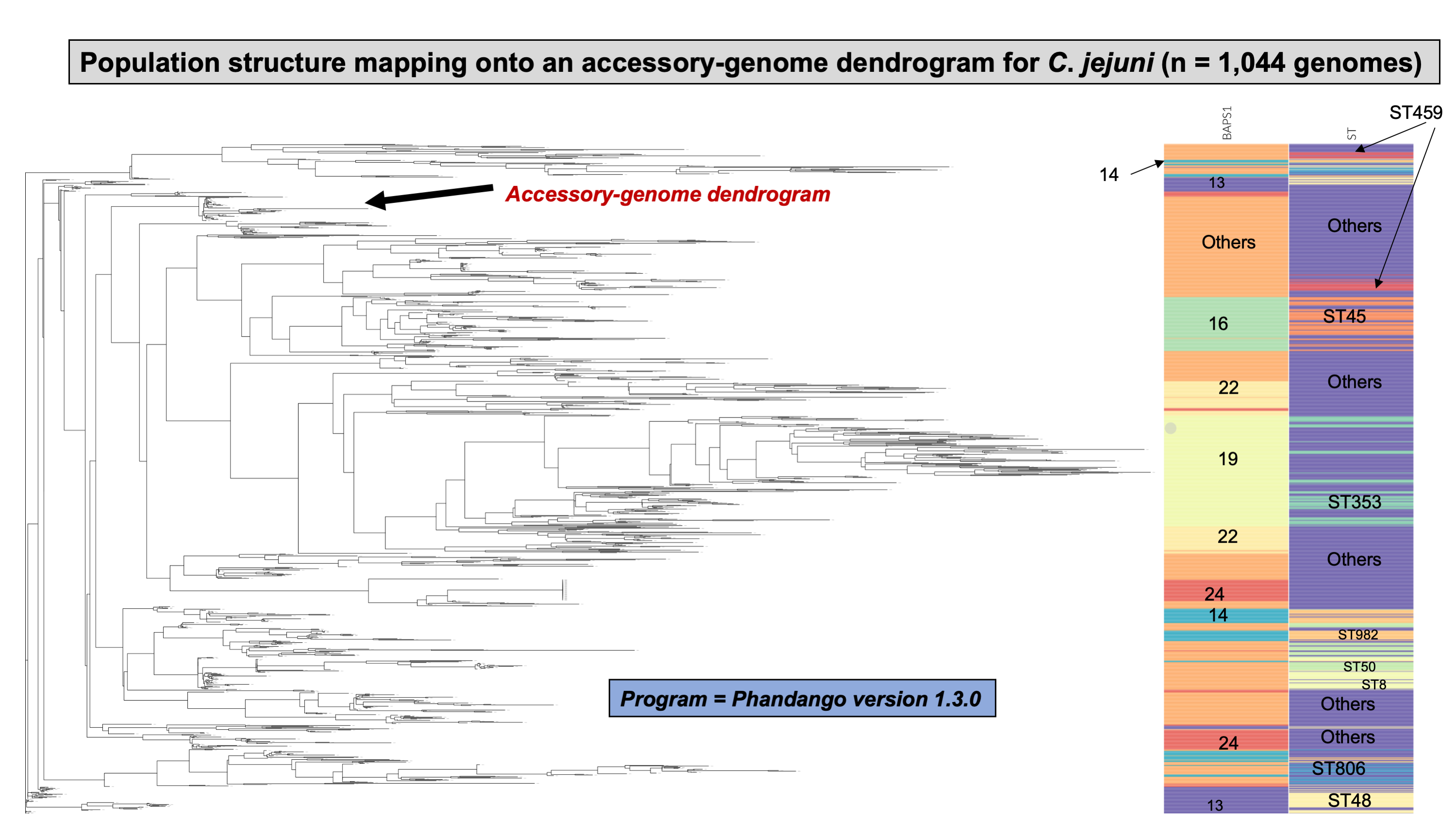

Supplement: Supplemental Information 5 — Accessory-genome file (binary matrix for gene presence and absence) was generated with Roary (.fa.newick file) and the population structure classification file was created using custom R scripts combining fastbaps (BAPS1 haplotypes or sub-groups) and MLST (STs) outputs. A total of 1,044 genomes were used for this analysis. Others indicate either other minor haplotypes/sub-groups or other STs for both BAPS1 and ST, respectively. This classification was done by grouping less representated variants into one group called “Others”. Plotting of the data was done with phandango v.1.3.0 to facilitate branch visualization. [file peerj-09-11376-s005.png]

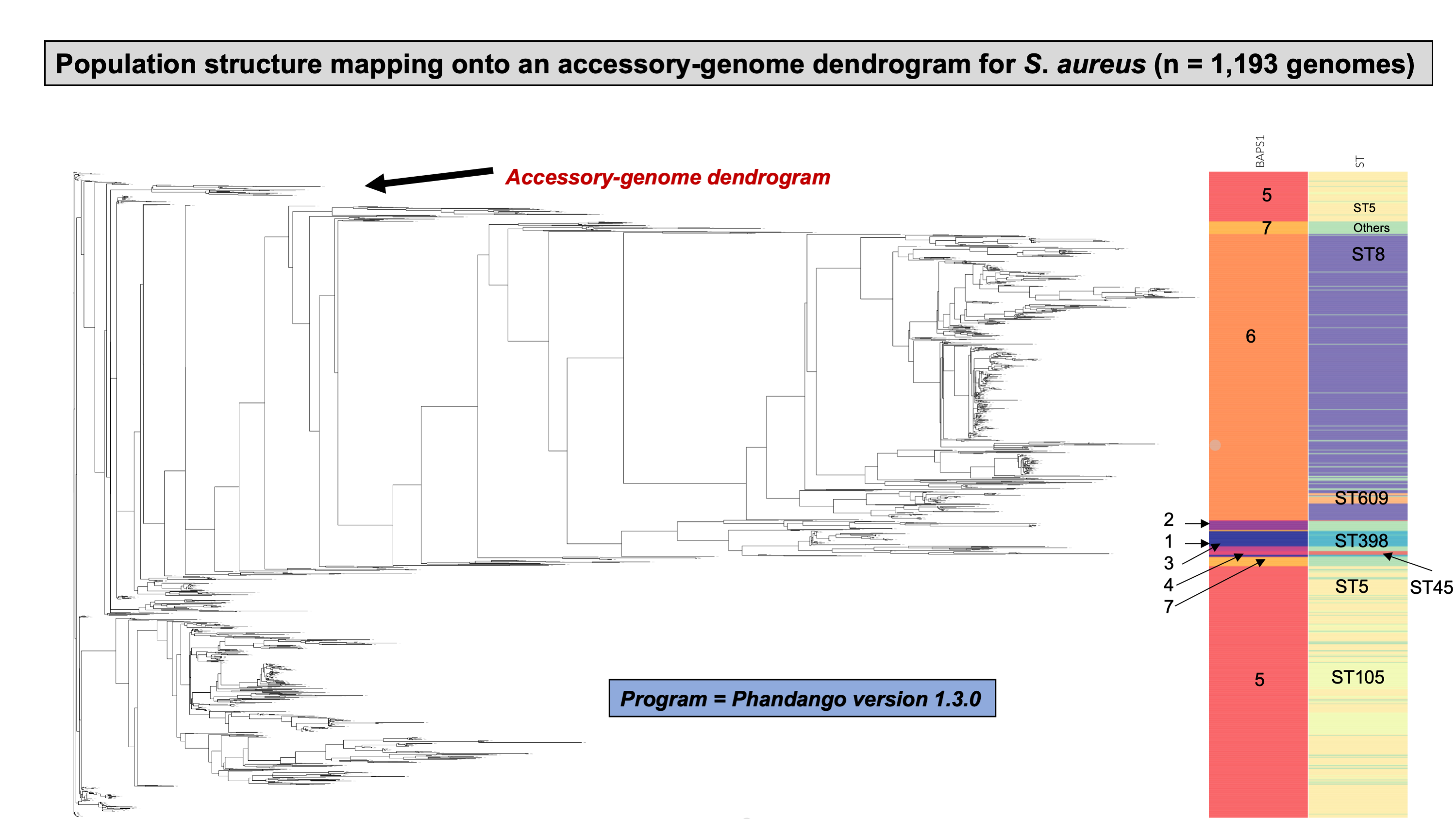

Supplement: Supplemental Information 6 — Accessory-genome file (binary matrix for gene presence and absence) was generated with Roary (.fa.newick file) and the population structure classification file was created using custom R scripts combining fastbaps (BAPS1 haplotypes or sub-groups) and MLST (STs) outputs. A total of 1,193 genomes were used for this analysis. Others indicate either other minor haplotypes/sub-groups or other STs for both BAPS1 and ST, respectively. This classification was done by grouping less represented variants into one group called “Others”. Plotting of the data was done with phandango v.1.3.0 to facilitate branch visualization. [file peerj-09-11376-s006.png]

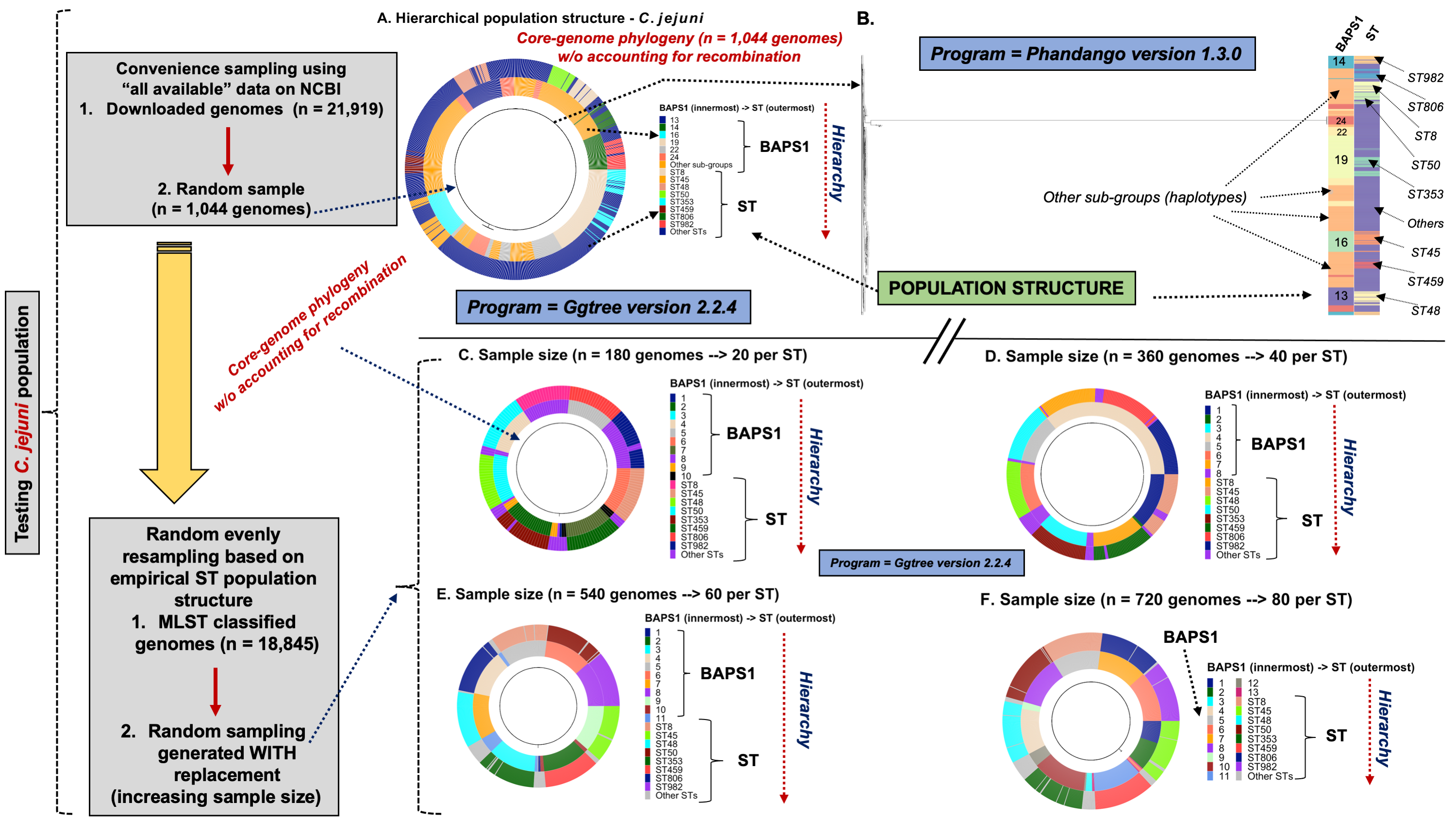

Supplement: Supplemental Information 7 — ’Initially, the C. jejuni population had 21,919 genomes downloaded from NCBI SRA (convenience sampling). A random sample of 1,044 genomes were used to analyze the empirical population structure and phylogenetic relationship between genomes. (A) Hierarchical population structure (BAPS1 haplotypes/sub-groups -> STs) of 1,044 C. jejuni genomes plotted onto a core-genome phylogeny. Visualization was accomplished with ggtree. (B) Similarly, to plot A, plot B uses the same data but plotting was done with phandango for comparison with ggtree. From 21,919 genomes, 18,845 genomes were classified with MLST into STs. Thereafter, a random sample generated with replacement was done to produce four datasets containing 180, 360, 540, and 720 of MLST-classified genomes. Sampling was done evenly across ST populations upon empirical determination of the population structure (considering only dominant STs and grouping minor ones into a single category called “Other STs”). A total of nine ST groups were ultimately used, and sampling was evenly distributed across them. (C) ggtree based visualization of 180 genomes using core-genome phylogeny and population structure mapping (20 genomes per ST group). (D) ggtree based visualization of 360 genomes using core-genome phylogeny and population structure mapping (40 genomes per ST group). (E) ggtree based visualization of 540 genomes using core-genome phylogeny and population structure mapping (60 genomes per ST group). (F) ggtree based visualization of 720 genomes using core-genome phylogeny and population structure mapping (80 genomes per ST group). Throughout the analysis, dominant BAPS1 sub-groups and STs were kept ungrouped; whereas, minor variants were aggregated into a single category called “Other sub-groups” or “Other STs” for BAPS1 and ST, respectively. Core-genome phylogenies were generated with FastTree analysis (w/o accounting for recombination) of core-genome alignments produced by Roary (.aln file). ggtree version 2.2.4 and phan [file peerj-09-11376-s007.png]

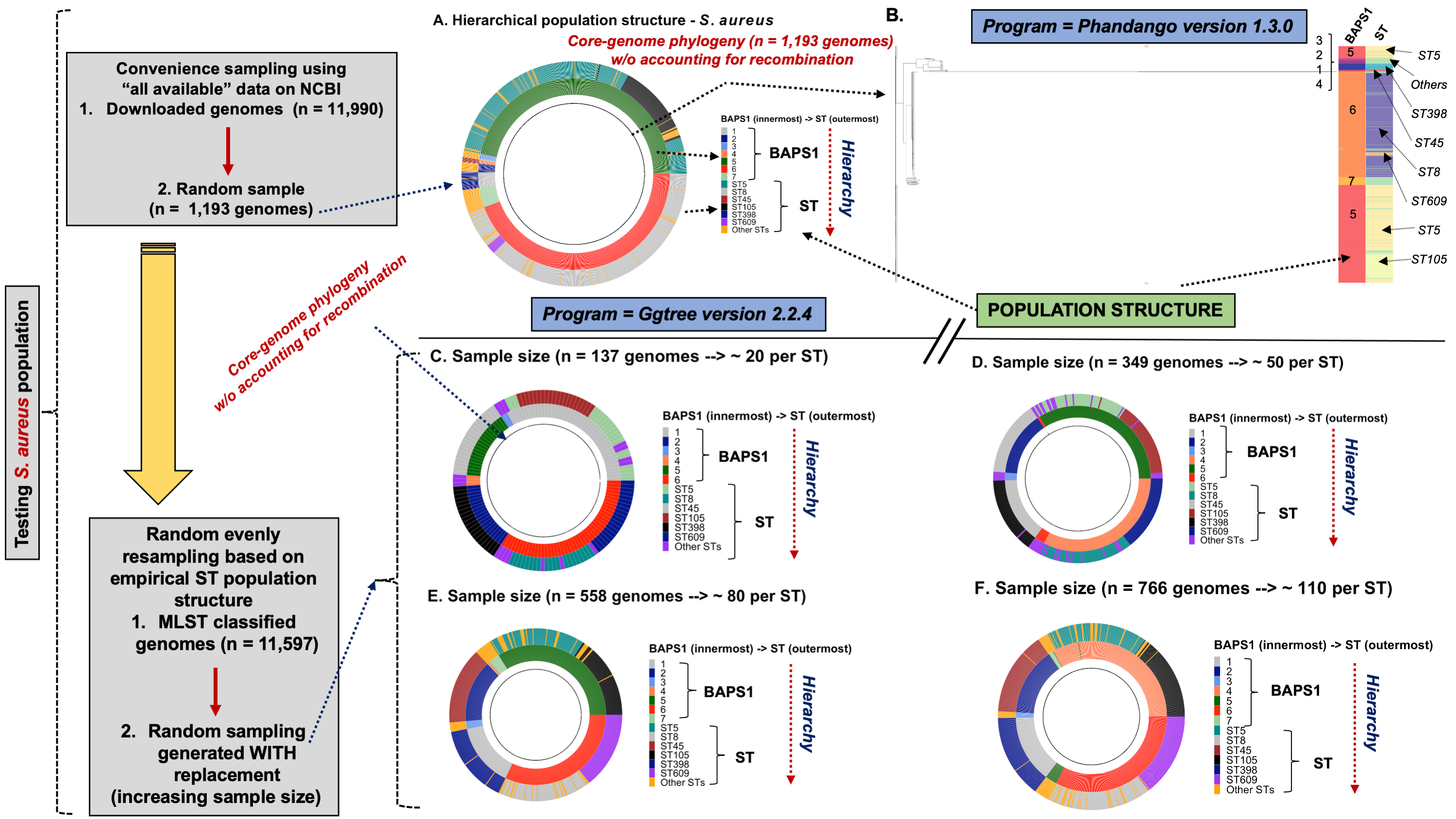

Supplement: Supplemental Information 8 — ’Initially, the S. aureus population had 11,990 genomes downloaded from NCBI SRA (convenience sampling). A random sample of 1,193 genomes were used to analyze the empirical population structure and phylogenetic relationship between genomes. (A) Hierarchical population structure (BAPS1 haplotypes/sub-groups -> STs) of 1,193 S. aureus genomes plotted onto a core-genome phylogeny. Visualization was accomplished with ggtree. (B) Similarly, to plot A, plot B uses the same data but plotting was done with phandango for comparison with ggtree. From 11,990 genomes, 11,597 genomes were classified with MLST into STs. Thereafter, a random sample generated with replacement was done to produce four datasets containing 140, 350, 560, and 770 of MLST-classified genomes. Sampling was done evenly across ST variants upon empirical determination of the population structure (considering only dominant STs and grouping minor ones into a single category called “Other STs”). A total of nine ST groups were ultimately used, and sampling was evenly distributed across them. (C) ggtree based visualization of 137 genomes using core-genome phylogeny and population structure mapping (~20 genomes per ST group-3 genomes belonging to the “Other STs” group did not pass quality control). (D) ggtree based visualization of 349 genomes using core-genome phylogeny and population structure mapping (~50 genomes per ST group-1 genomes belonging to the “Other STs” group did not pass quality control). (E) ggtree based visualization of 558 genomes using core-genome phylogeny and population structure mapping (~80 genomes per ST group-2 genomes belonging to the “Other STs” group did not pass quality control). (F) ggtree based visualization of 766 genomes using core-genome phylogeny and population structure mapping (~110 genomes per ST group-4 genomes belonging to the “Other STs” group did not pass quality control). Throughout the analysis, dominant BAPS1 sub-groups and STs were kept ungrouped; whereas, minor varian [file peerj-09-11376-s008.png]

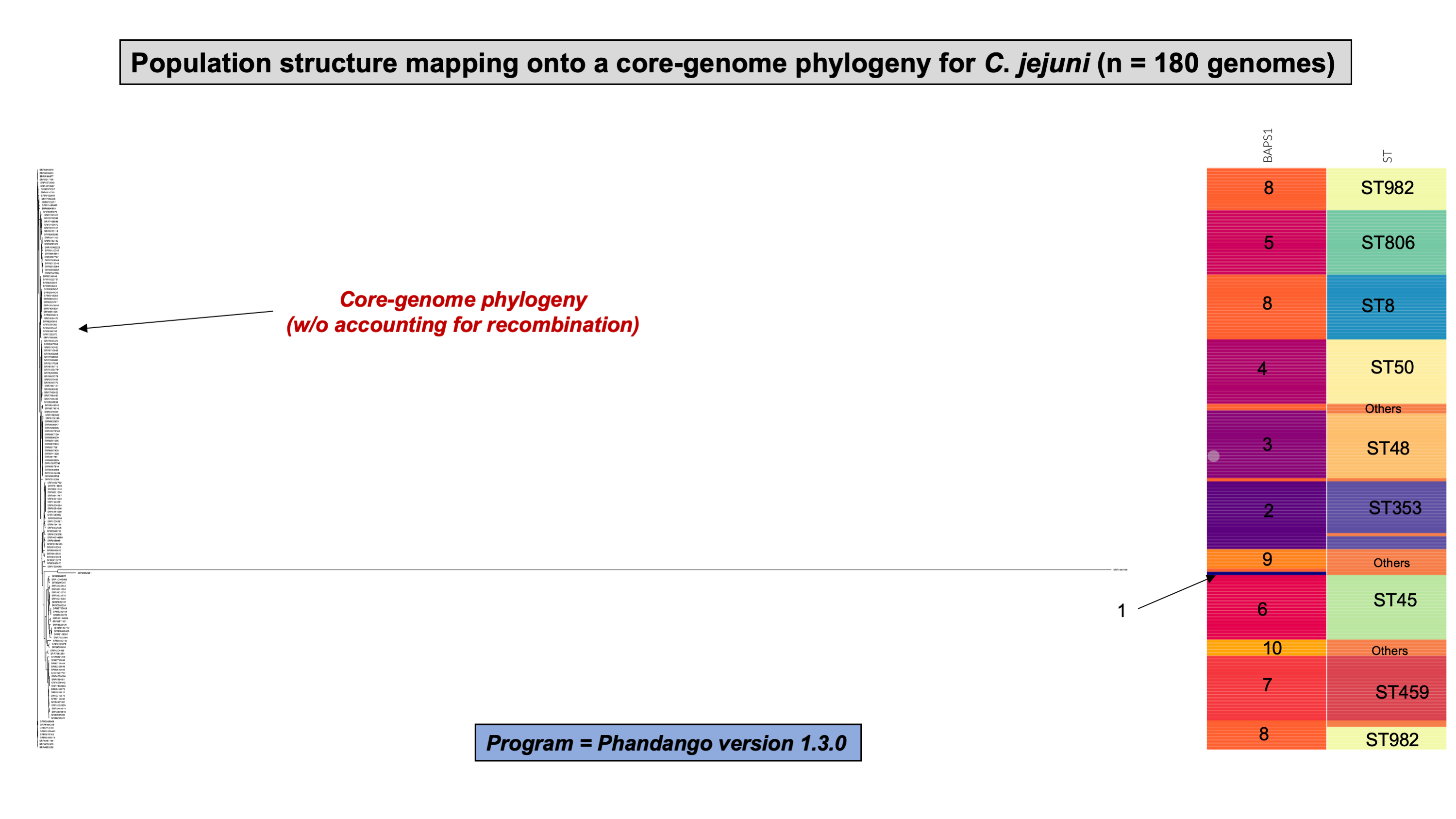

Supplement: Supplemental Information 9 — Core-genome alignment was generated with Roary (.aln file), phylogeny (w/o accounting for recombination) was done using FastTree, and the population structure classification file was created using custom R scripts combining fastbaps (BAPS1 haplotypes/sub-groups) and MLST (STs) outputs. A total of 180 genomes were randomly selected from a population of 18,845 MLST-classified genomes for this analysis. Genomes were evenly sampled across ST populations (20 per ST group), upon considering the ST-based empirical population structure of C. jejuni, which comprised the following dominant and minor variants: ST8, ST45, ST48, ST50, ST353, ST459, ST806, ST982, and the minor ones combined as Others (“Other STs”). There were ten distinct BAPS1 haplotypes or sub-groups, and there was no need for sub-grouping into “Others” for minor representatives. Plotting of the data was done with phandango v.1.3.0 to facilitate branch visualization. [file peerj-09-11376-s009.png]

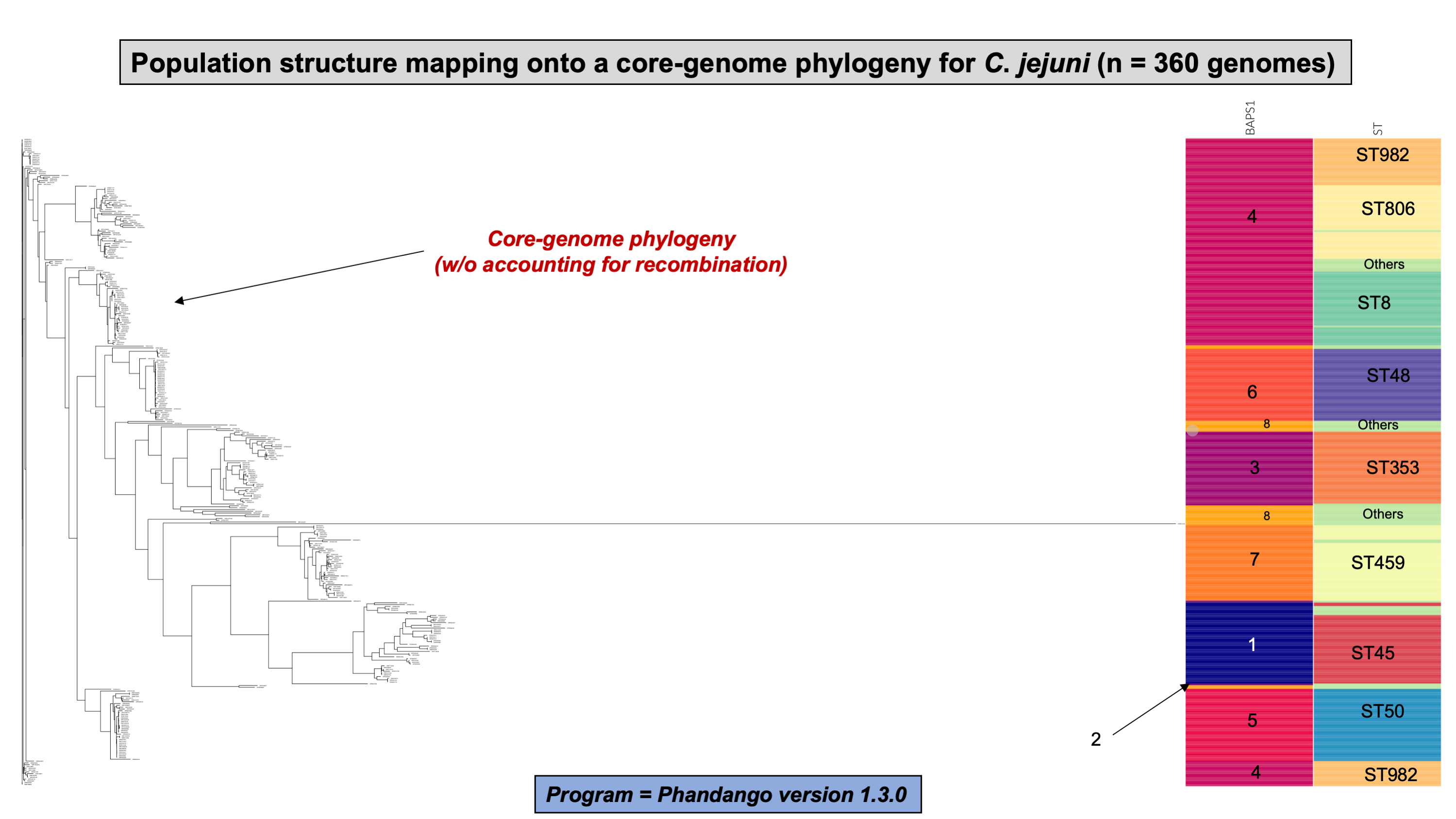

Supplement: Supplemental Information 10 — Core-genome alignment was generated with Roary (.aln file), phylogeny (w/o accounting for recombination) was done using FastTree, and the population structure classification file was created using custom R scripts combining fastbaps (BAPS1 haplotypes/sub-groups) and MLST (STs) outputs. A total of 360 genomes were randomly selected from a population of 18,845 MLST-classified genomes for this analysis. Genomes were evenly sampled across ST populations (40 per ST group), upon considering the ST-based empirical population structure of C. jejuni, which comprised the following dominant and minor variants: ST8, ST45, ST48, ST50, ST353, ST459, ST806, ST982, and the minor ones combined as Others (“Other STs”). There were eight distinct BAPS1 haplotypes or sub-groups, and there was no need for sub-grouping into “Others” for minor representatives. Plotting of the data was done with phandango v.1.3.0 to facilitate branch visualization. [file peerj-09-11376-s010.png]

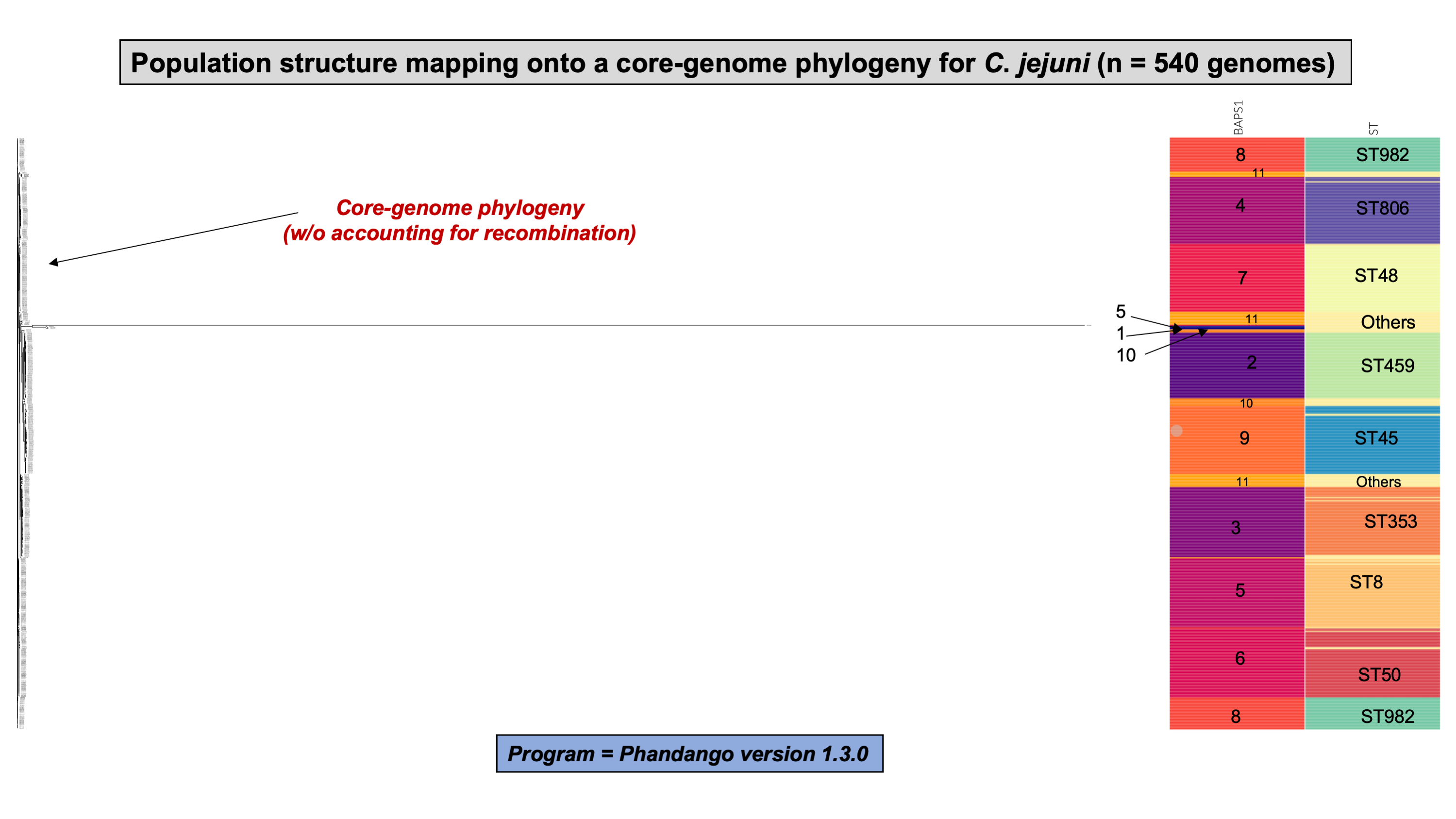

Supplement: Supplemental Information 11 — Core-genome alignment was generated with Roary (.aln file), phylogeny (w/o accounting for recombination) was done using FastTree, and the population structure classification file was created using custom R scripts combining fastbaps (BAPS1 haplotypes/sub-groups) and MLST (STs) outputs. A total of 540 genomes were randomly selected from a population of 18,845 MLST-classified genomes for this analysis. Genomes were evenly sampled across ST populations (60 per ST group), upon considering the ST-based empirical population structure of C. jejuni, which comprised the following dominant and minor variants: ST8, ST45, ST48, ST50, ST353, ST459, ST806, ST982, and the minor ones combined as Others (“Other STs”). There were eleven distinct BAPS1 haplotypes or sub-groups, and there was no need for sub-grouping into “Others” for minor representatives. Plotting of the data was done with phandango v.1.3.0 to facilitate branch visualization. [file peerj-09-11376-s011.png]

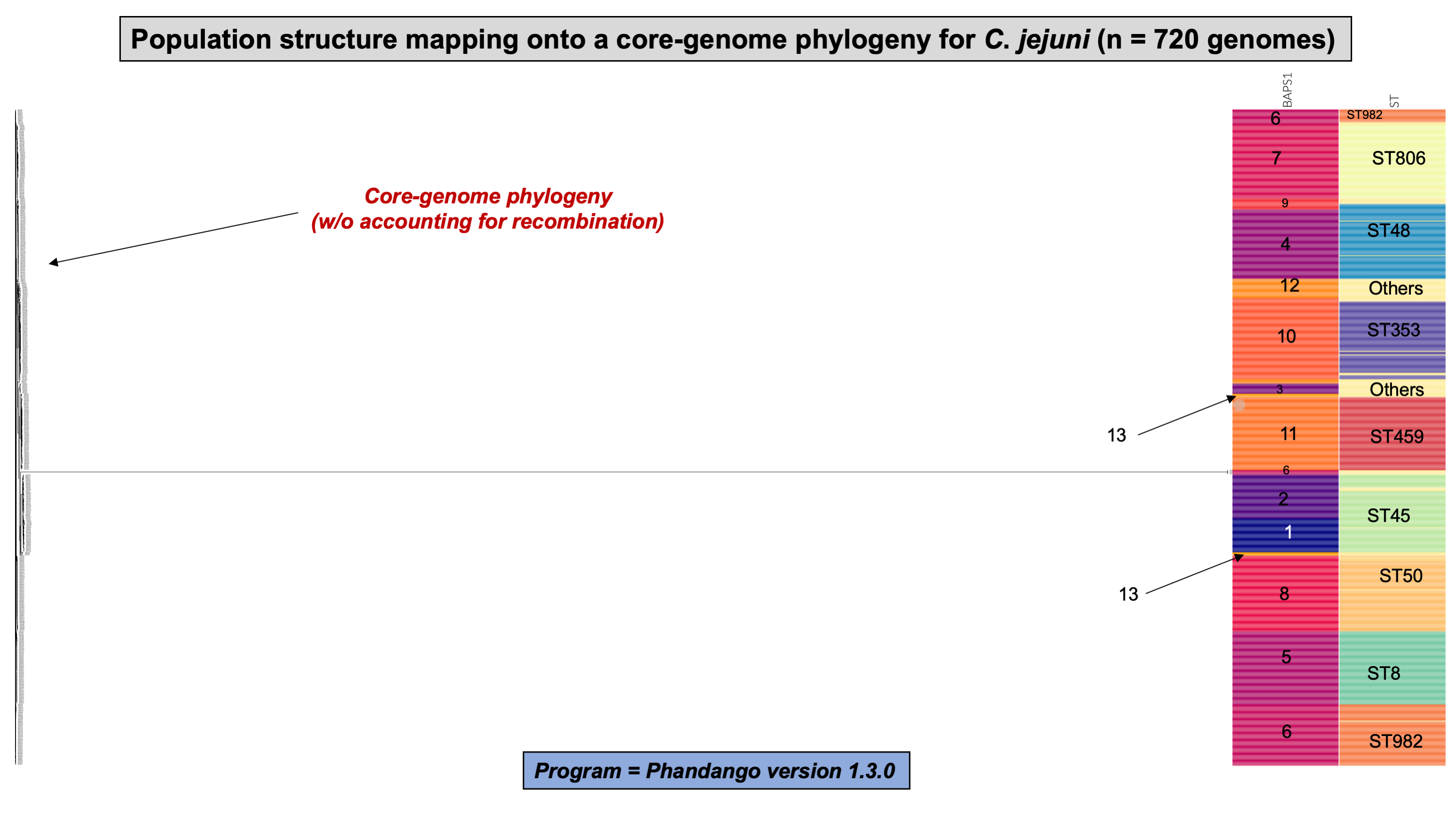

Supplement: Supplemental Information 12 — Core-genome alignment was generated with Roary (.aln file), phylogeny (w/o accounting for recombination) was done using FastTree, and the population structure classification file was created using custom R scripts combining fastbaps (BAPS1 haplotypes/sub-groups) and MLST (STs) outputs. A total of 720 genomes were randomly selected from a population of 18,845 MLST-classified genomes for this analysis. Genomes were evenly sampled across ST populations (80 per ST group), upon considering the ST-based empirical population structure of C. jejuni, which comprised the following dominant and minor variants: ST8, ST45, ST48, ST50, ST353, ST459, ST806, ST982, and the minor ones combined as Others (“Other STs”). There were twelve distinct BAPS1 haplotypes or sub-groups, and there was no need for sub-grouping into “Others” for minor representatives. Plotting of the data was done with phandango v.1.3.0 to facilitate branch visualization. [file peerj-09-11376-s012.png]

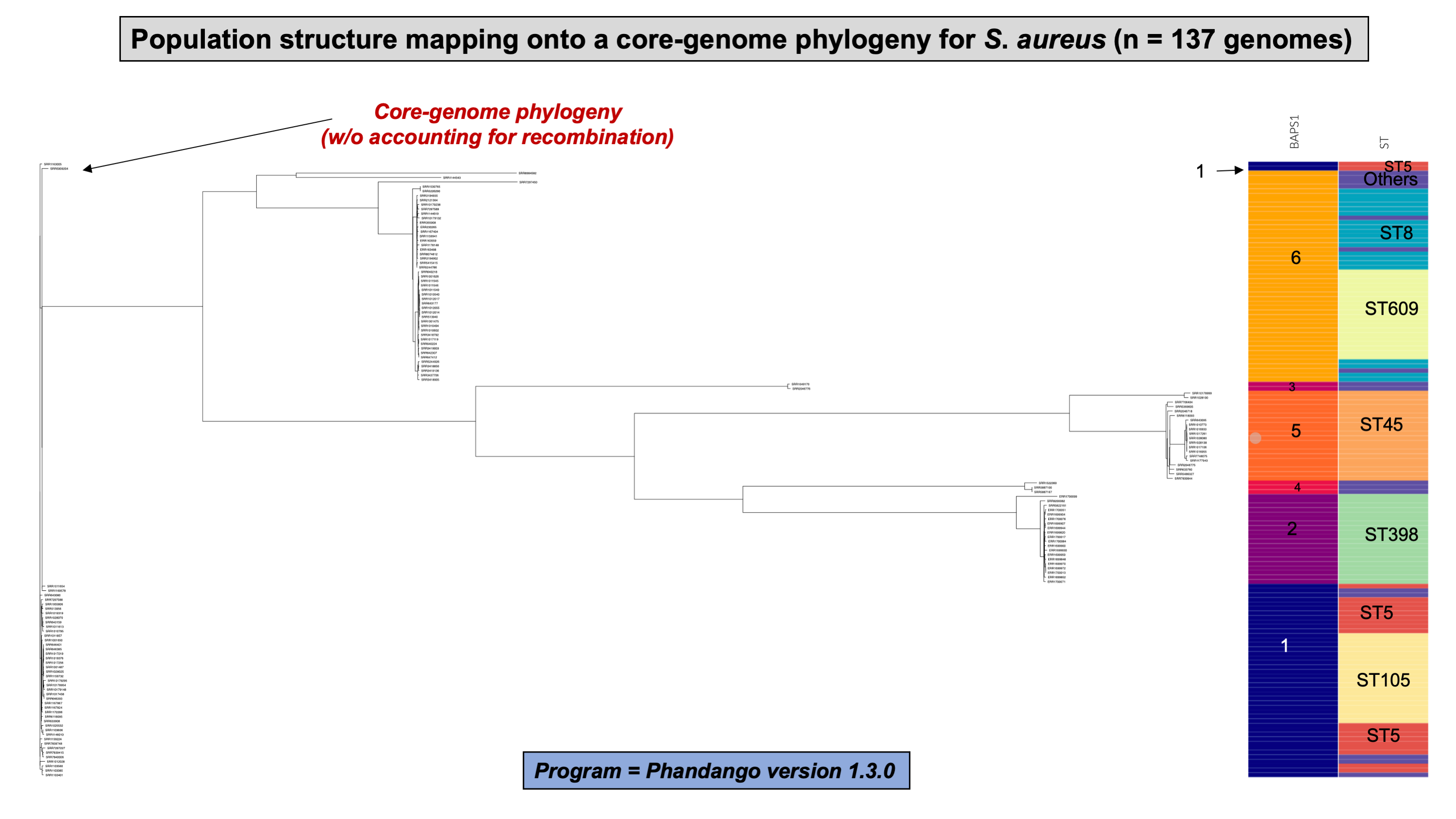

Supplement: Supplemental Information 13 — Core-genome alignment was generated with Roary (.aln file), phylogeny (w/o accounting for recombination) was done using FastTree, and the population structure classification file was created using custom R scripts combining fastbaps (BAPS1 haplotypes/sub-groups) and MLST (STs) outputs. A total of 140 genomes were randomly selected from a population of 11,597 MLST-classified genomes for this analysis. Genomes were evenly sampled across ST populations (20 per ST group), upon considering the ST-based empirical population structure of C. jejuni, which comprised the following dominant and minor variants: ST5, ST8, ST45, ST105, ST398, ST609, and the minor ones combined as Others (“Other STs”). There were six distinct BAPS1 haplotypes or sub-groups, and there was no need for sub-grouping into “Others” for minor representatives. At the end a total of 137 genomes were used for plotting, because three (belonged to “Other STs” group) did not yield high-quality haplotypes in the core-genome alignment. Plotting of the data was done with phandango v.1.3.0 to facilitate branch visualization. [file peerj-09-11376-s013.png]

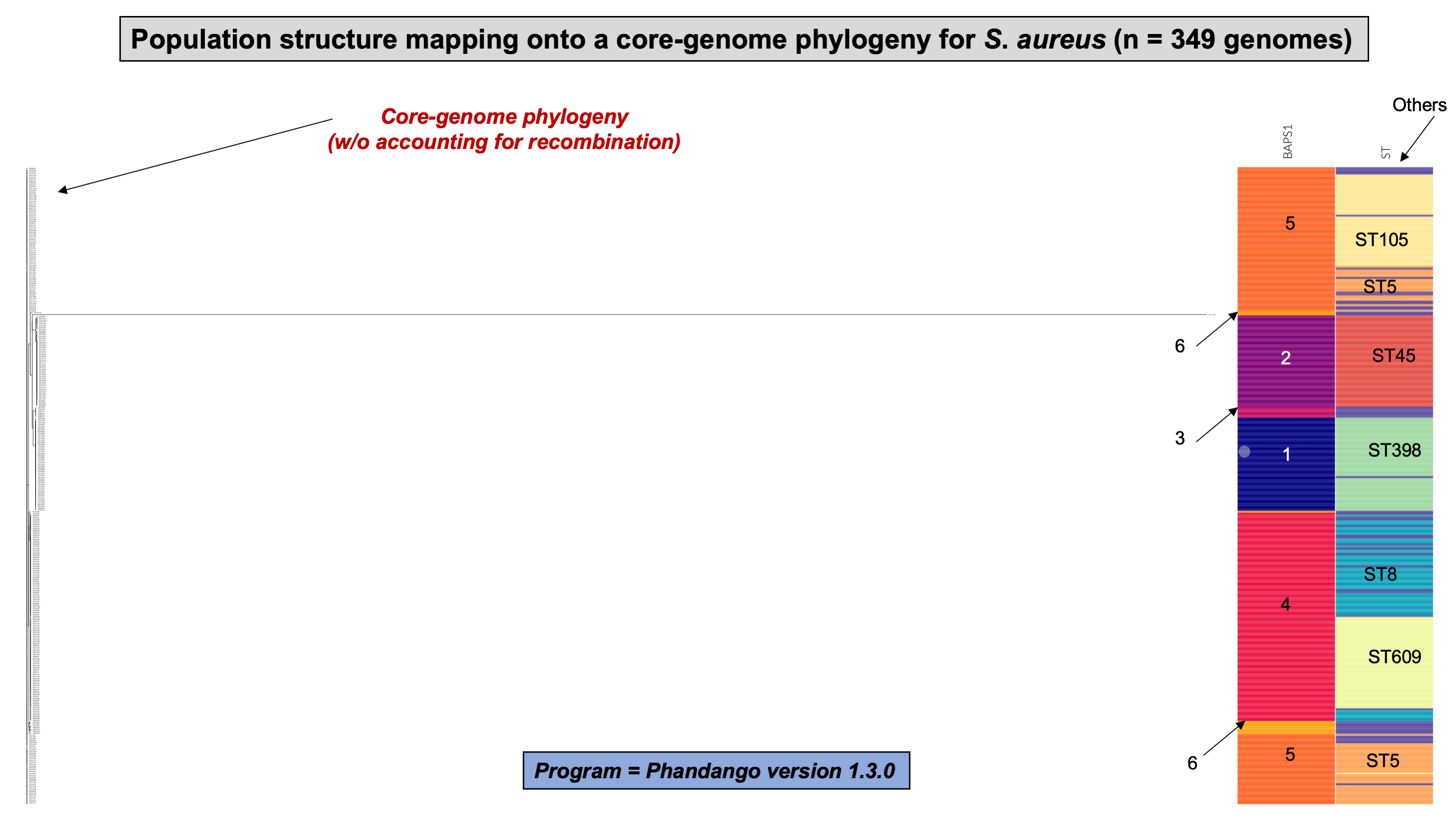

Supplement: Supplemental Information 14 — Core-genome alignment was generated with Roary (.aln file), phylogeny (w/o accounting for recombination) was done using FastTree, and the population structure classification file was created using custom R scripts combining fastbaps (BAPS1 haplotypes/sub-groups) and MLST (STs) outputs. A total of 350 genomes were randomly selected from a population of 11,597 MLST-classified genomes for this analysis. Genomes were evenly sampled ST populations (50 per ST group), upon considering the ST-based empirical population structure of C. jejuni, which comprised the following dominant and minor variants: ST5, ST8, ST45, ST105, ST398, ST609, and the minor ones combined as Others (“Other STs”). There were six distinct BAPS1 haplotypes or sub-groups, and there was no need for sub-grouping into “Others” for minor representatives. At the end a total of 349 genomes were used for plotting, because one (belonged to “Other STs” group) did not yield high-quality haplotypes in the core-genome alignment. Plotting of the data was done with phandango v.1.3.0 to facilitate branch visualization. [file peerj-09-11376-s014.png]

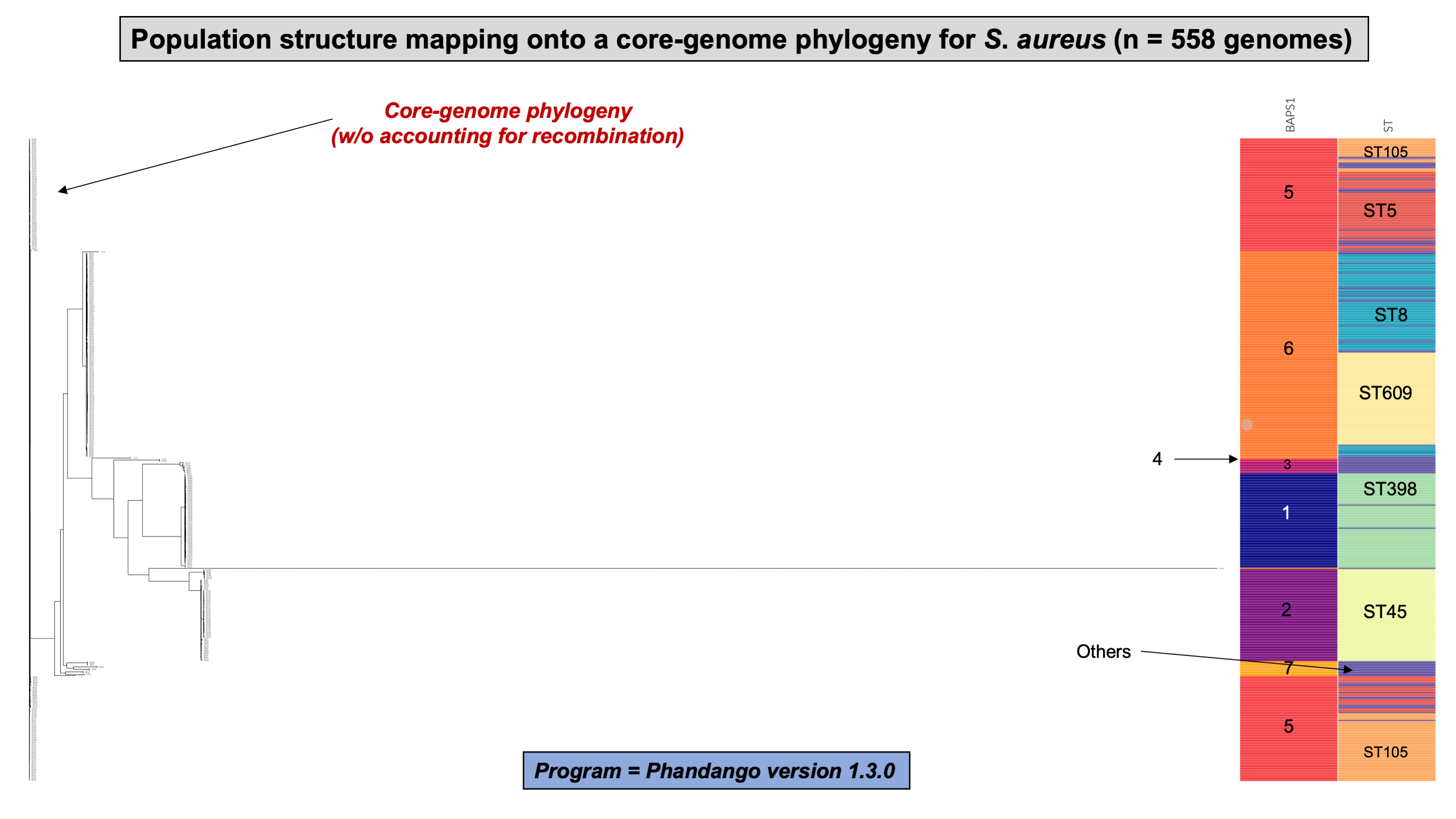

Supplement: Supplemental Information 15 — Core-genome alignment was generated with Roary (.aln file), phylogeny (w/o accounting for recombination) was done using FastTree, and the population structure classification file was created using custom R scripts combining fastbaps (BAPS1 haplotypes/sub-groups) and MLST (STs) outputs. A total of 560 genomes were randomly selected from a population of 11,597 MLST-classified genomes for this analysis. Genomes were evenly sampled ST populations (80 per ST group), upon considering the ST-based empirical population structure of C. jejuni, which comprised the following dominant and minor variants: ST5, ST8, ST45, ST105, ST398, ST609, and the minor ones combined as Others (“Other STs”). There were seven distinct BAPS1 haplotypes or sub-groups, and there was no need for sub-grouping into “Others” for minor representatives. At the end a total of 558 genomes were used for plotting, because two (belonged to “Other STs” group) did not yield high-quality haplotypes in the core-genome alignment. Plotting of the data was done with phandango v.1.3.0 to facilitate branch visualization. [file peerj-09-11376-s015.png]

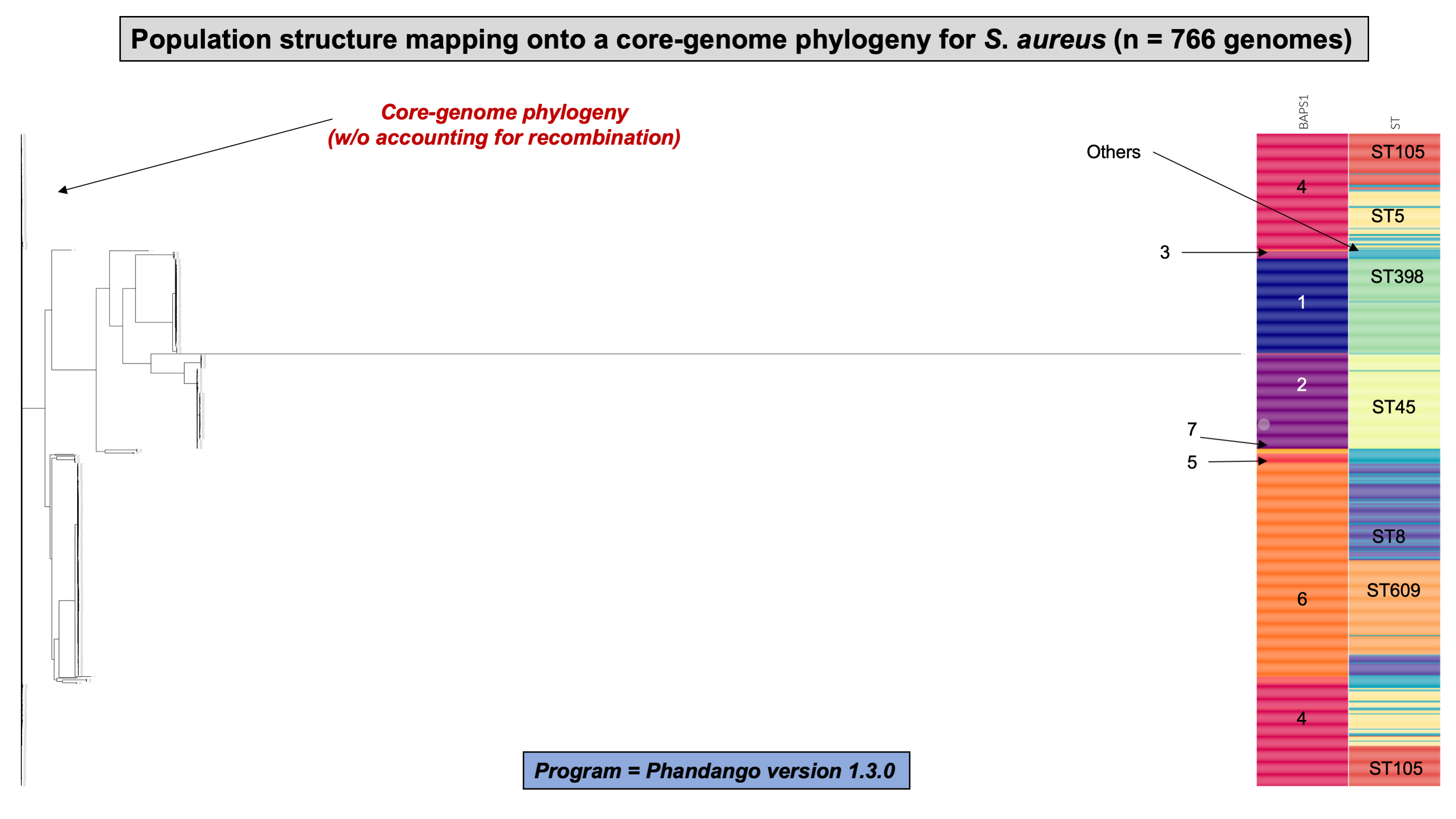

Supplement: Supplemental Information 16 — Core-genome alignment was generated with Roary (.aln file), phylogeny (w/o accounting for recombination) was done using FastTree, and the population structure classification file was created using custom R scripts combining fastbaps (BAPS1 haplotypes/sub-groups) and MLST (STs) outputs. A total of 770 genomes were randomly selected from a population of 11,597 MLST-classified genomes for this analysis. Genomes were evenly sampled across ST populations (110 per ST group), upon considering the ST-based empirical population structure of C. jejuni, which comprised the following dominant and minor variants: ST5, ST8, ST45, ST105, ST398, ST609, and the minor ones combined as Others (“Other STs”). There were seven distinct BAPS1 haplotypes or sub-groups, and there was no need for sub-grouping into “Others” for minor representatives. At the end a total of 766 genomes were used for plotting, because four (belonged to “Other STs” group) did not yield high-quality haplotypes in the core-genome alignment. Plotting of the data was done with phandango v.1.3.0 to facilitate branch visualization. [file peerj-09-11376-s016.png]
